# Supplementary material for: Proteasome subunit PSMC3 variants cause neurosensory syndrome combining deafness and cataract due to proteotoxic stress
Source: EMBO Mol Med. 2020 Jun 5;12(7):e11861. doi: 10.15252/emmm.201911861 (PMC7338805; doi:10.15252/emmm.201911861)
Supplement: Supplementary file 2 — Movie EV1 [file EMMM-12-e11861-s002.zip › Movie_EV1.docx]

**
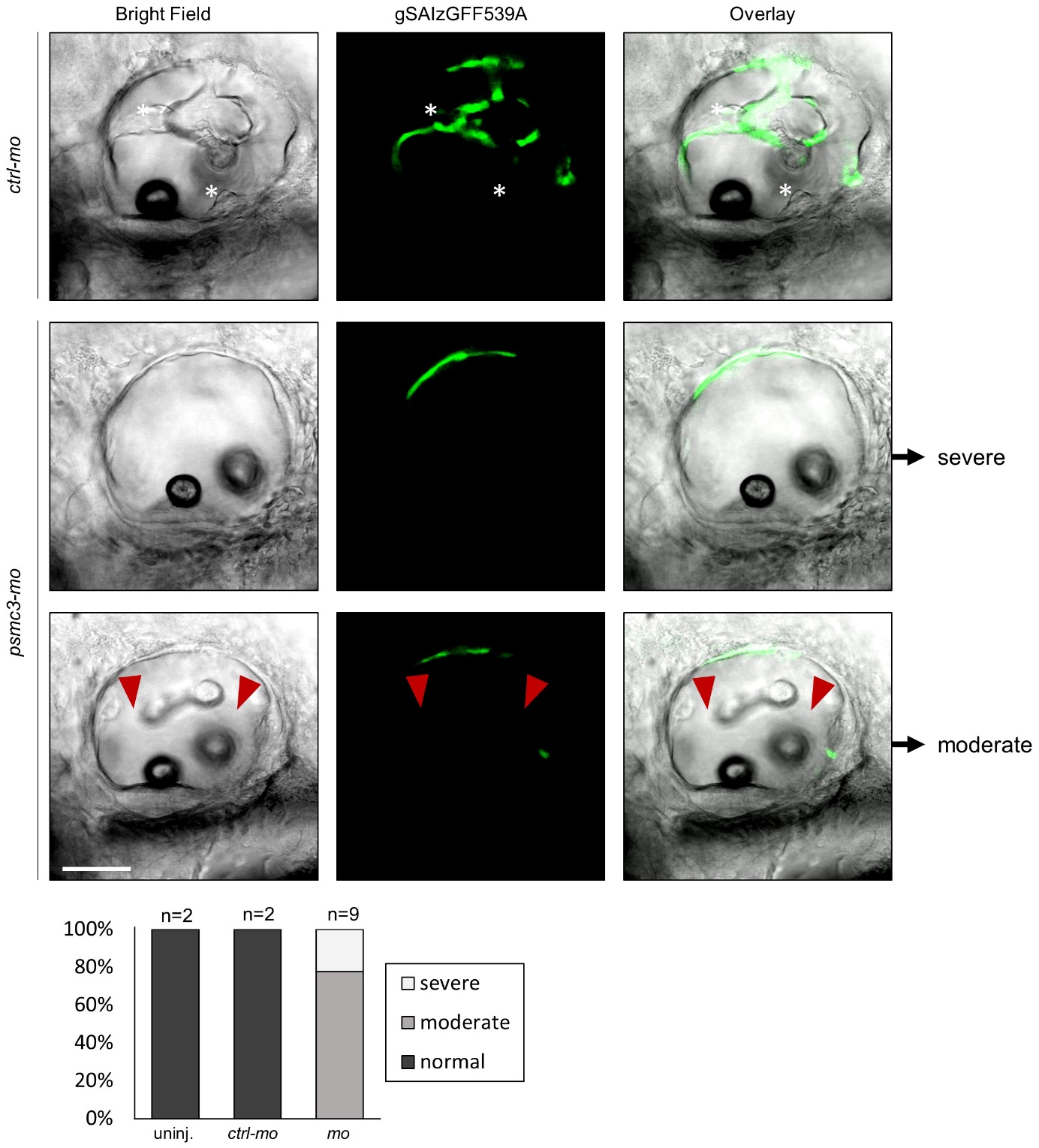
**

**Movie EV1. Life imaging of semicircular canal formation (56 and 72 hpf) in *ctrl-mo* and *mo* injected embryos.**

Morpholino (MO)-mediated knock-down of Psmc3 resulted in abnormal ear development. Canal pillars form between 56-72 hpf in uninjected and control injected fish (*ctrl-mo*) but not in morphants (*mo*). White asterisks indicate fused pillars. Red arrowheads mark unfused projections. Severe = no outgrowth of projections of semicircular canals. Moderate = projections do not fuse. Scale bar = 50 µm.

**Supplementary references**

Adzhubei, I.A., S. Schmidt, L. Peshkin, V.E. Ramensky, A. Gerasimova, P. Bork, A.S. Kondrashov, and S.R. Sunyaev. 2010. A method and server for predicting damaging missense mutations. *Nat. Methods*. 7:248–249. doi:10.1038/nmeth0410-248.

Binato, R., C.E. Alvarez Martinez, L. Pizzatti, B. Robert, and E. Abdelhay. 2006. SMAD 8 binding to mice <em>Msx1</em> basal promoter is required for transcriptional activation. *Biochem. J.* 393:141. doi:10.1042/BJ20050327.

Etard, C., S. Joshi, J. Stegmaier, R. Mikut, and U. Strähle. 2017. Tracking of Indels by DEcomposition is a Simple and Effective Method to Assess Efficiency of Guide RNAs in Zebrafish. *Zebrafish*. 14:586–588. doi:10.1089/zeb.2017.1454.

Geng, F.-S., L. Abbas, S. Baxendale, C.J. Holdsworth, A.G. Swanson, K. Slanchev, M. Hammerschmidt, J. Topczewski, and T.T. Whitfield. 2013. Semicircular canal morphogenesis in the zebrafish inner ear requires the function of gpr126 (lauscher), an adhesion class G protein-coupled receptor gene. *Development*. 140:4362–4374. doi:10.1242/dev.098061.

Geoffroy, V., C. Pizot, C. Redin, A. Piton, N. Vasli, C. Stoetzel, A. Blavier, J. Laporte, and J. Muller. 2015. VaRank: a simple and powerful tool for ranking genetic variants. *PeerJ*. 3:e796. doi:10.7717/peerj.796.

Guerrero, C., T. Milenković, N. Pržulj, P. Kaiser, and L. Huang. 2008. Characterization of the proteasome interaction network using a QTAX-based tag-team strategy and protein interaction network analysis. *Proc. Natl. Acad. Sci.* 105:13333. doi:10.1073/pnas.0801870105.

Kumar, P., S. Henikoff, and P.C. Ng. 2009. Predicting the effects of coding non-synonymous variants on protein function using the SIFT algorithm. *Nat. Protoc.* 4:1073–1081. doi:10.1038/nprot.2009.86.

Ogris, C., D. Guala, M. Kaduk, and E.L.L. Sonnhammer. 2017. FunCoup 4: new species, data, and visualization. *Nucleic Acids Res.* 46:D601–D607. doi:10.1093/nar/gkx1138.

O’Leary, N.A., M.W. Wright, J.R. Brister, S. Ciufo, D. Haddad, R. McVeigh, B. Rajput, B. Robbertse, B. Smith-White, D. Ako-Adjei, A. Astashyn, A. Badretdin, Y. Bao, O. Blinkova, V. Brover, V. Chetvernin, J. Choi, E. Cox, O. Ermolaeva, C.M. Farrell, T. Goldfarb, T. Gupta, D. Haft, E. Hatcher, W. Hlavina, V.S. Joardar, V.K. Kodali, W. Li, D. Maglott, P. Masterson, K.M. McGarvey, M.R. Murphy, K. O’Neill, S. Pujar, S.H. Rangwala, D. Rausch, L.D. Riddick, C. Schoch, A. Shkeda, S.S. Storz, H. Sun, F. Thibaud-Nissen, I. Tolstoy, R.E. Tully, A.R. Vatsan, C. Wallin, D. Webb, W. Wu, M.J. Landrum, A. Kimchi, T. Tatusova, M. DiCuccio, P. Kitts, T.D. Murphy, and K.D. Pruitt. 2016. Reference sequence (RefSeq) database at NCBI: current status, taxonomic expansion, and functional annotation. *Nucleic Acids Res.* 44:D733–D745. doi:10.1093/nar/gkv1189.

Perkins, D.N., D.J. Pappin, D.M. Creasy, and J.S. Cottrell. 1999. Probability-based protein identification by searching sequence databases using mass spectrometry data. *Electrophoresis*. 20:3551–3567. doi:10.1002/(SICI)1522-2683(19991201)20:18<3551::AID-ELPS3551>3.0.CO;2-2.

Reese, M.G., F.H. Eeckman, D. Kulp, and D. Haussler. 1997. Improved Splice Site Detection in Genie. *J. Comput. Biol.* 4:311–323. doi:10.1089/cmb.1997.4.311.

Ruzicka, L., D.G. Howe, S. Ramachandran, S. Toro, C.E. Van Slyke, Y.M. Bradford, A. Eagle, D. Fashena, K. Frazer, P. Kalita, P. Mani, R. Martin, S.T. Moxon, H. Paddock, C. Pich, K. Schaper, X. Shao, A. Singer, and M. Westerfield. 2018. The Zebrafish Information Network: new support for non-coding genes, richer Gene Ontology annotations and the Alliance of Genome Resources. *Nucleic Acids Res.* 47:D867–D873. doi:10.1093/nar/gky1090.

Scott, A.F., C.A. Bocchini, J.S. Amberger, and A. Hamosh. 2018. OMIM.org: leveraging knowledge across phenotype–gene relationships. *Nucleic Acids Res.* 47:D1038–D1043. doi:10.1093/nar/gky1151.

Shapiro, M.B., and P. Senapathy. 1987. RNA splice junctions of different classes of eukaryotes: sequence statistics and functional implications in gene expression. *Nucleic Acids Res.* 15:7155–7174.

The UniProt Consortium. 2016. UniProt: the universal protein knowledgebase. *Nucleic Acids Res.* 45:D158–D169. doi:10.1093/nar/gkw1099.

Thorvaldsdóttir, H., J.T. Robinson, and J.P. Mesirov. 2013. Integrative Genomics Viewer (IGV): high-performance genomics data visualization and exploration. *Brief. Bioinform.* 14:178–192. doi:10.1093/bib/bbs017.

Wang, Y., X. Zhang, H. Zhang, Y. Lu, H. Huang, X. Dong, J. Chen, J. Dong, X. Yang, H. Hang, and T. Jiang. 2012. Coiled-coil networking shapes cell molecular machinery. *Mol. Biol. Cell*. 23:3911–3922. doi:10.1091/mbc.e12-05-0396.

Yeo, G., and C.B. Burge. 2004. Maximum entropy modeling of short sequence motifs with applications to RNA splicing signals. *J. Comput. Biol. J. Comput. Mol. Cell Biol.* 11:377–394. doi:10.1089/1066527041410418.

Zerbino, D.R., P. Achuthan, W. Akanni, M.R. Amode, D. Barrell, J. Bhai, K. Billis, C. Cummins, A. Gall, C.G. Girón, L. Gil, L. Gordon, L. Haggerty, E. Haskell, T. Hourlier, O.G. Izuogu, S.H. Janacek, T. Juettemann, J.K. To, M.R. Laird, I. Lavidas, Z. Liu, J.E. Loveland, T. Maurel, W. McLaren, B. Moore, J. Mudge, D.N. Murphy, V. Newman, M. Nuhn, D. Ogeh, C.K. Ong, A. Parker, M. Patricio, H.S. Riat, H. Schuilenburg, D. Sheppard, H. Sparrow, K. Taylor, A. Thormann, A. Vullo, B. Walts, A. Zadissa, A. Frankish, S.E. Hunt, M. Kostadima, N. Langridge, F.J. Martin, M. Muffato, E. Perry, M. Ruffier, D.M. Staines, S.J. Trevanion, B.L. Aken, F. Cunningham, A. Yates, and P. Flicek. 2017. Ensembl 2018. *Nucleic Acids Res.* 46:D754–D761. doi:10.1093/nar/gkx1098.
