# Supplementary figures and images for: Proteasome subunit PSMC3 variants cause neurosensory syndrome combining deafness and cataract due to proteotoxic stress
Source: EMBO Mol Med. 2020 Jun 5;12(7):e11861. doi: 10.15252/emmm.201911861 (PMC7338805; doi:10.15252/emmm.201911861)

Appendix Figure S7

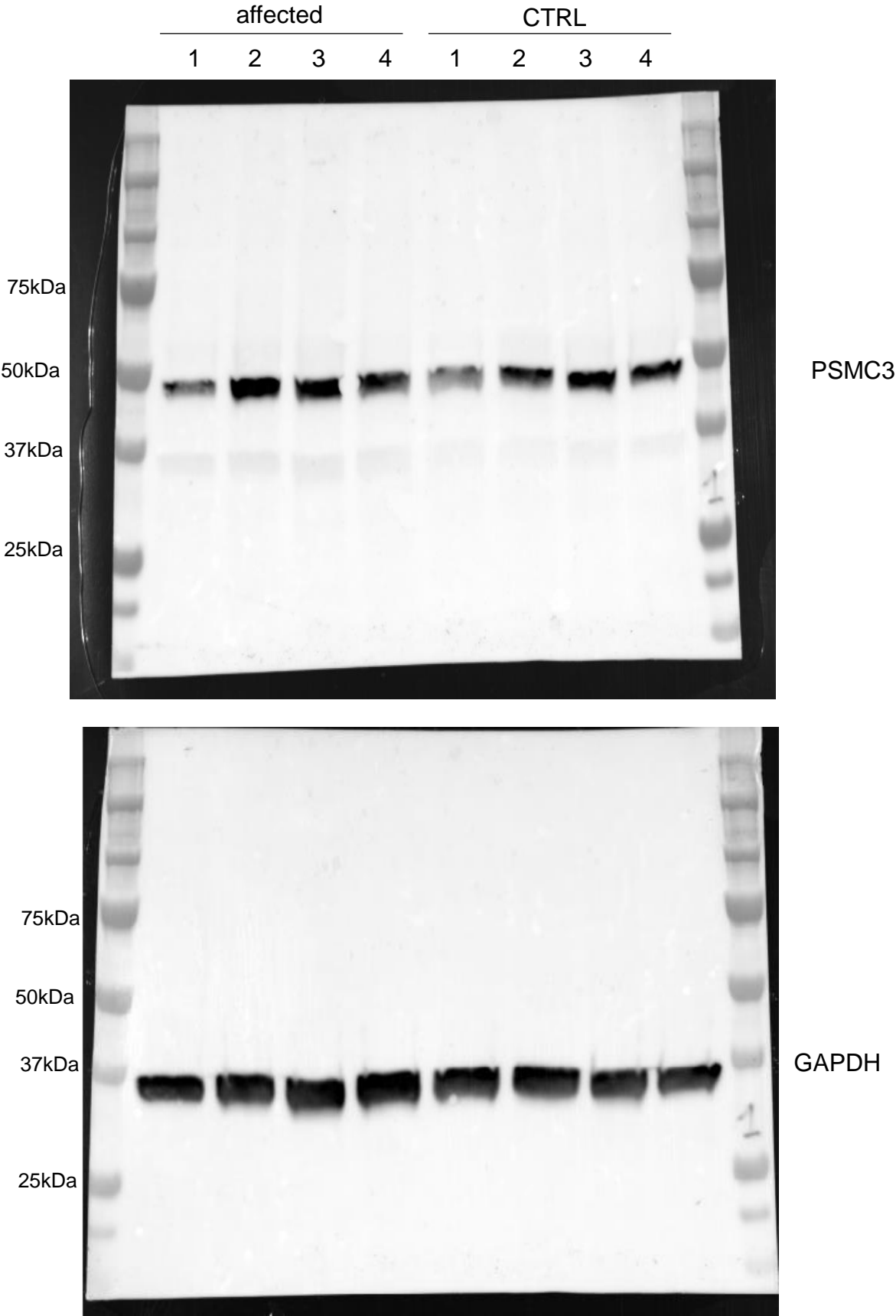

Supplement: Supplementary file 3 — Source Data for Appendix [file EMMM-12-e11861-s007.zip › Appendix_Figure_S7.pdf]

Appendix Figure S5C

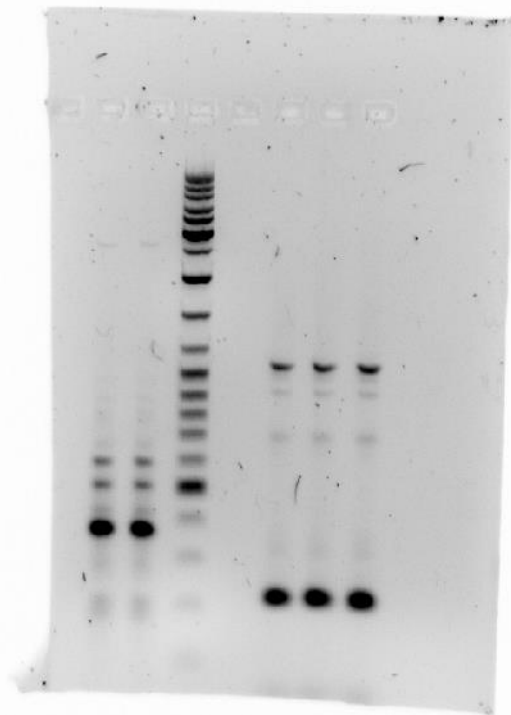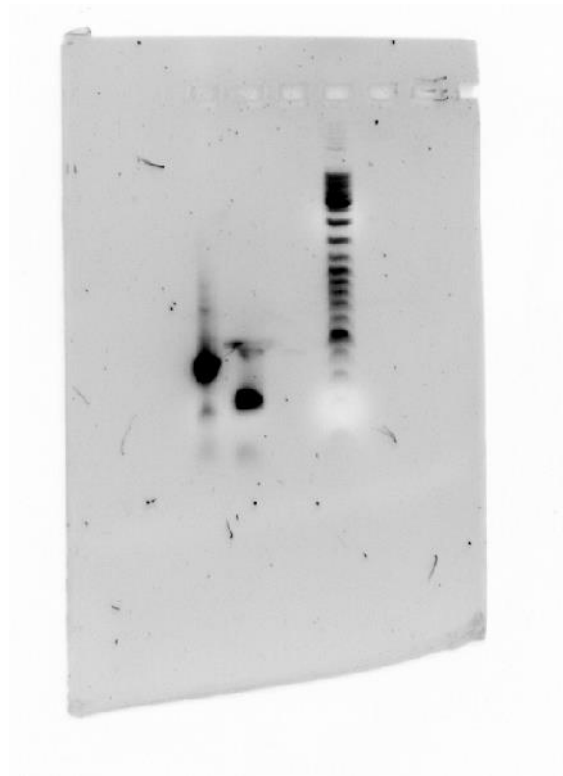

Supplement: Supplementary file 3 — Source Data for Appendix [file EMMM-12-e11861-s007.zip › Appendix_Figure_S5C.pdf]

Figure 1E

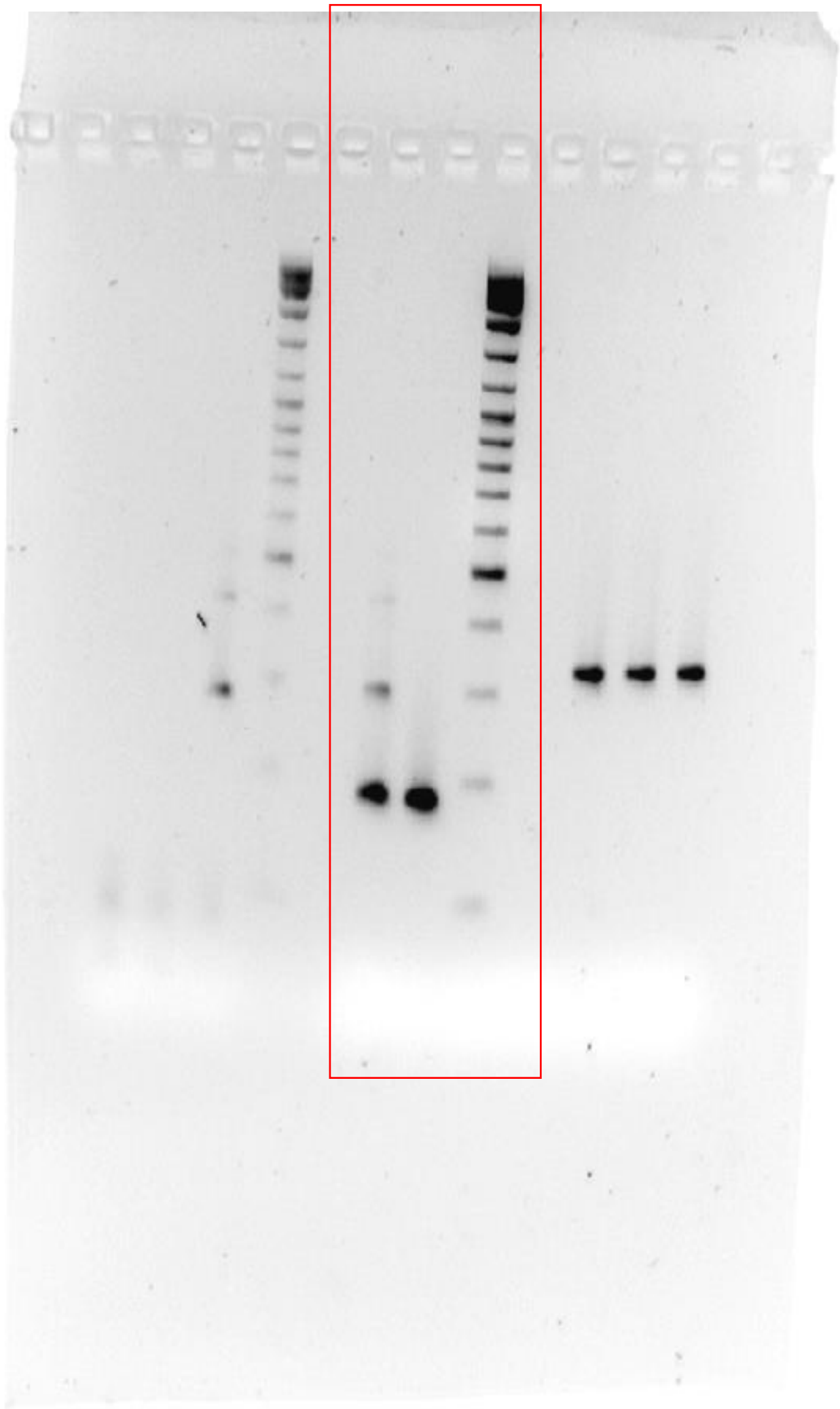

Supplement: Supplementary file 5 — Source Data for Figure 1 [file EMMM-12-e11861-s003.pdf]
