# Supplementary material for: Proteasome subunit PSMC3 variants cause neurosensory syndrome combining deafness and cataract due to proteotoxic stress
Source: EMBO Mol Med. 2020 Jun 5;12(7):e11861. doi: 10.15252/emmm.201911861 (PMC7338805; doi:10.15252/emmm.201911861)

Figure 3A

Suc-LLVA-AMC activity:

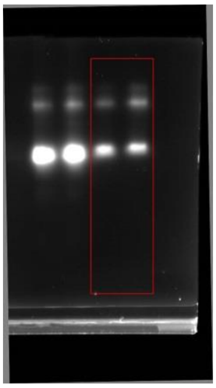

Figure 3C

Alpha6 western blot:

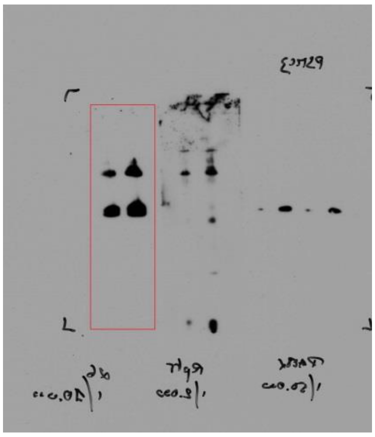

PSMC3 western blot:

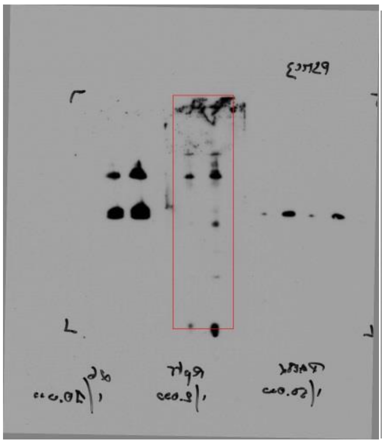

PA28-α western blot:

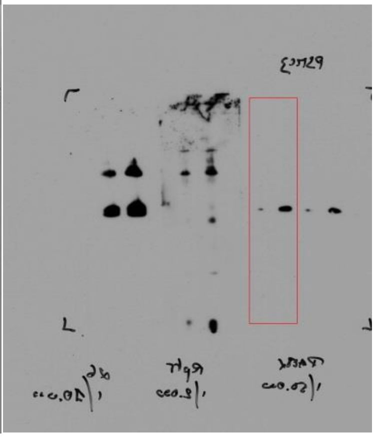

Figure 3D

Ubiquitin western blot:

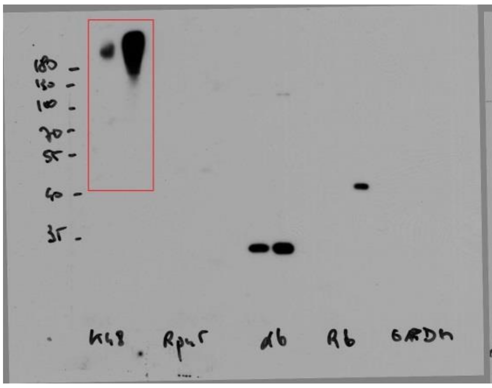

Alpha6 western blot:

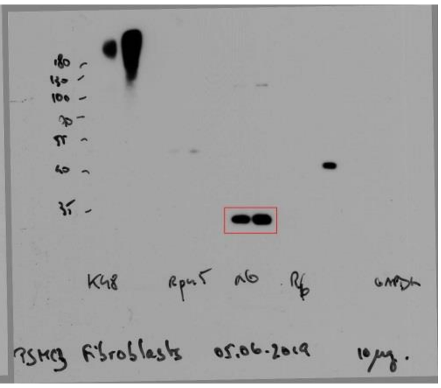

Beta1 western blot:

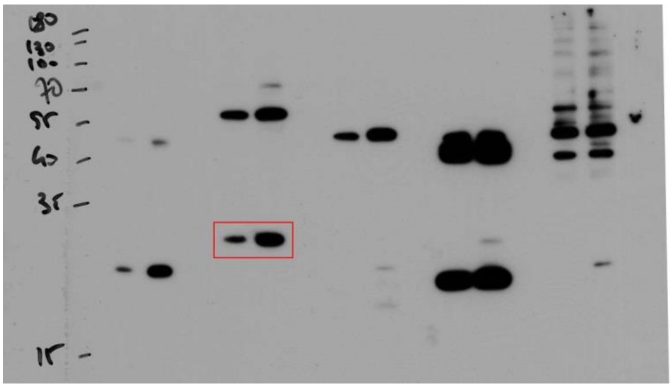

Beta2 western blot:

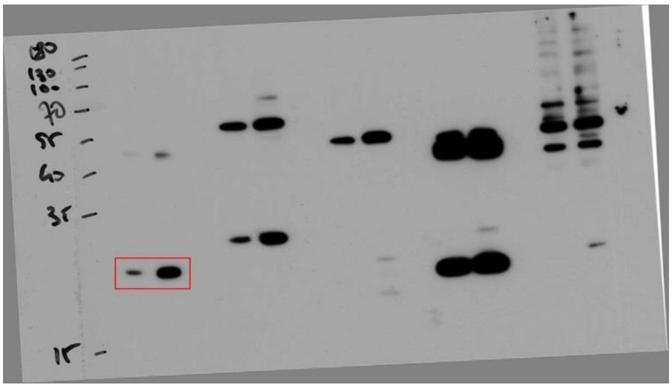

Beta5 western blot:

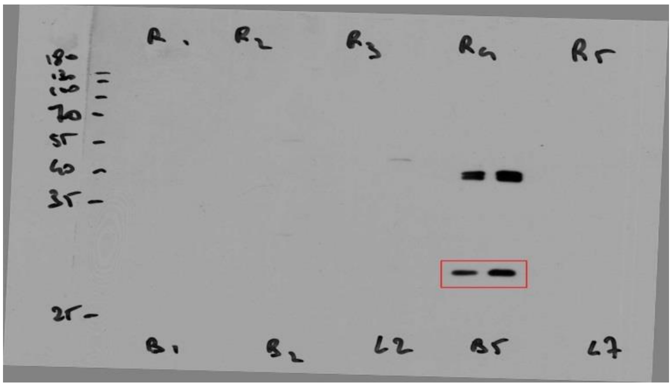

Beta5i western blot:

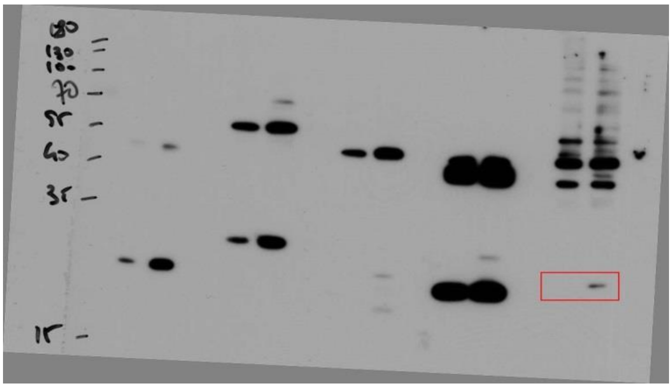

PSMC1 western blot:

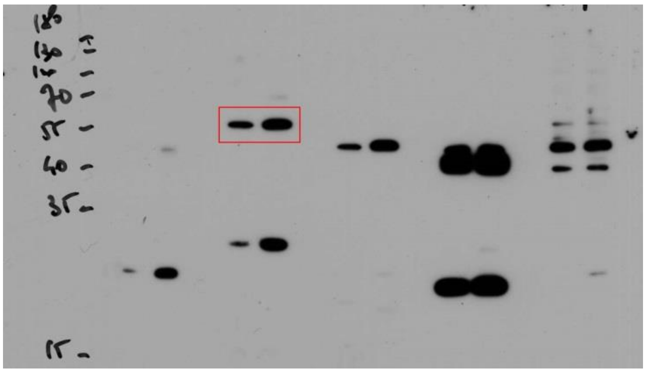

PSMC3 western blot (short exposure)

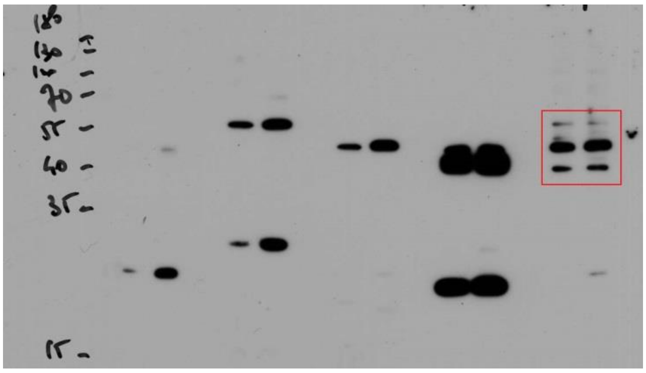

PSMC3 western blot (long exposure)

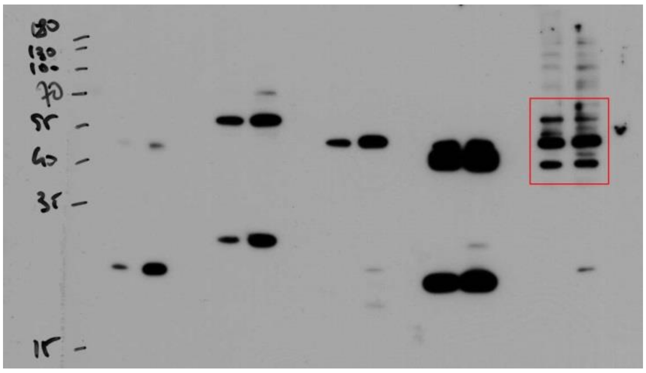

PSMC4 western blot:

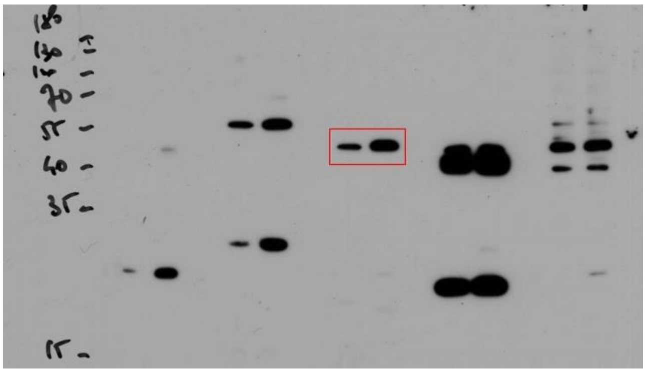

PSMC6 western blot:

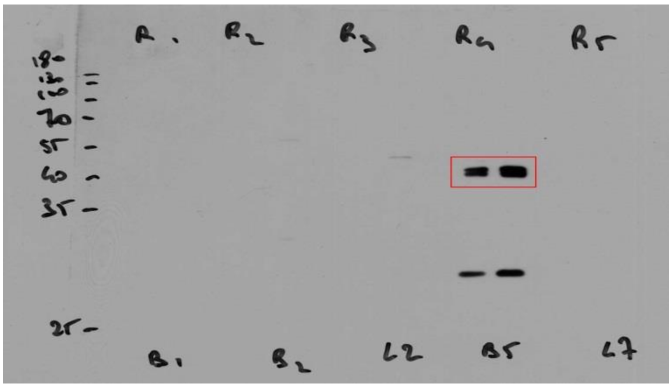

PA28-α western blot:

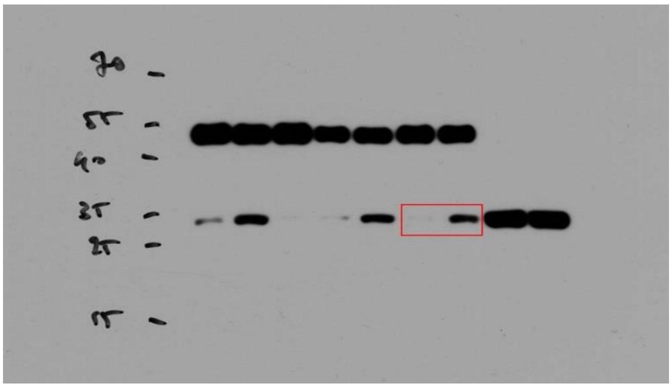

Tubulin western-blot:

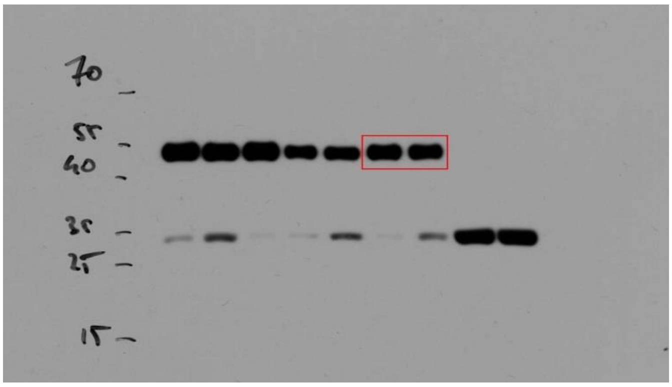

Supplement: Supplementary file 6 — Source Data for Figure 3 [file EMMM-12-e11861-s004.pdf]
