# Supplementary material for: Proteasome subunit PSMC3 variants cause neurosensory syndrome combining deafness and cataract due to proteotoxic stress
Source: EMBO Mol Med. 2020 Jun 5;12(7):e11861. doi: 10.15252/emmm.201911861 (PMC7338805; doi:10.15252/emmm.201911861)

Figure 4

Ubiquitin western blot

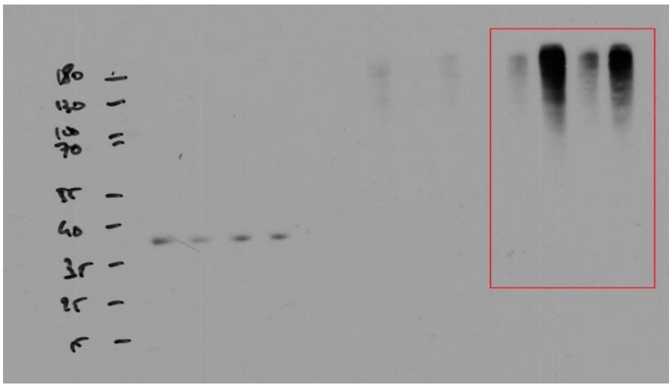

TCF11/Nrf1 western blot (short exposure):

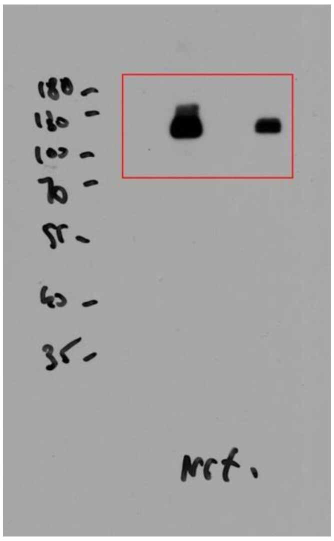

TCF11/Nrf1 western blot (long exposure):

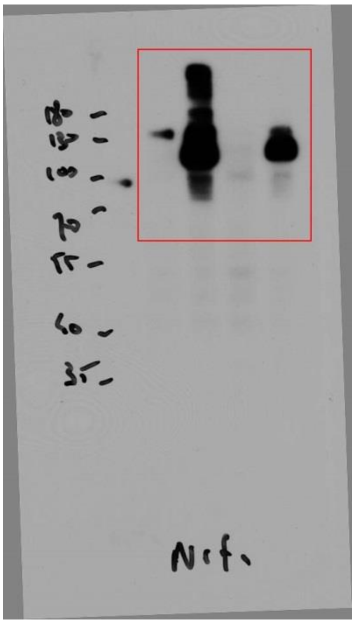

PSMC2 western blot:

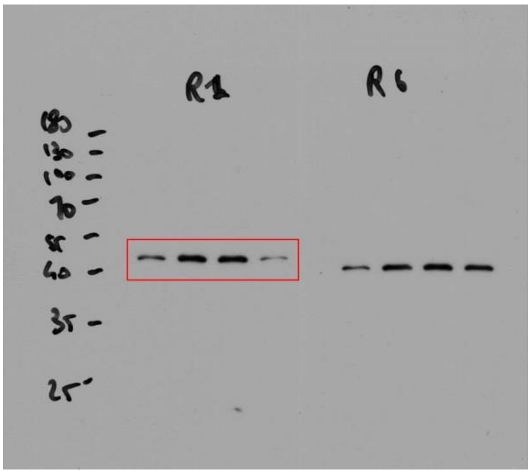

PSMC5 western blot:

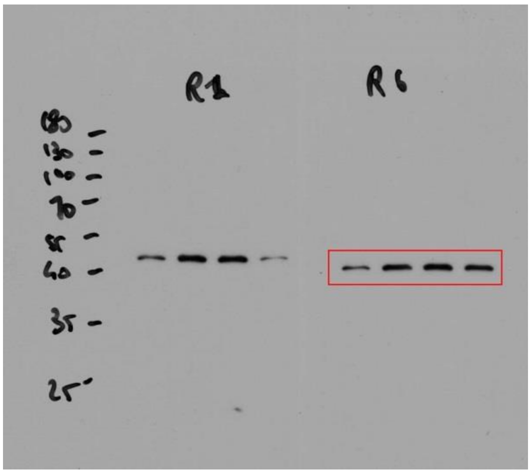

PSMC3 western blot:

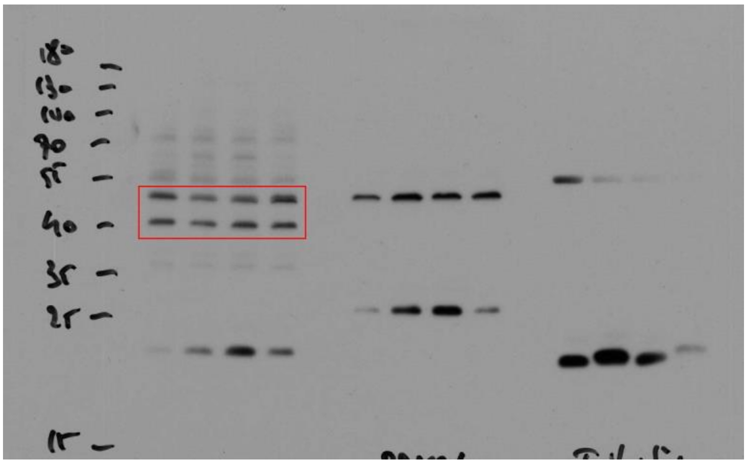

PSMC4 western blot:

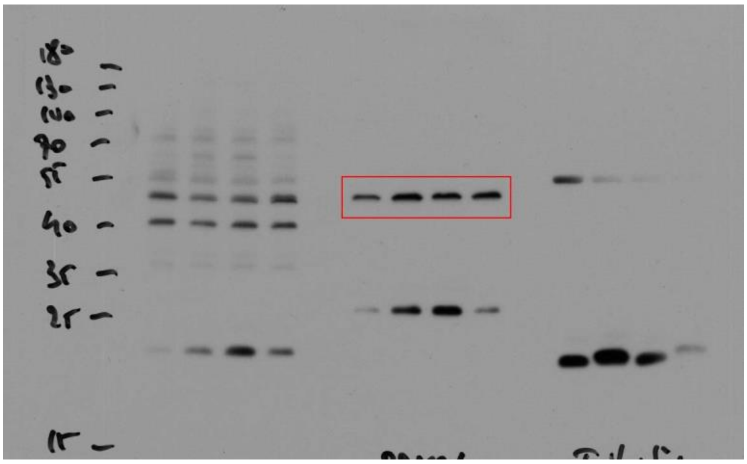

Beta1 western blot:

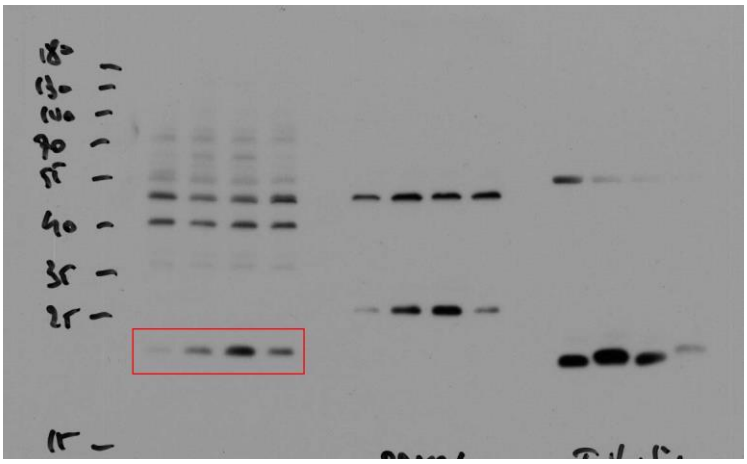

Beta2 western blot:

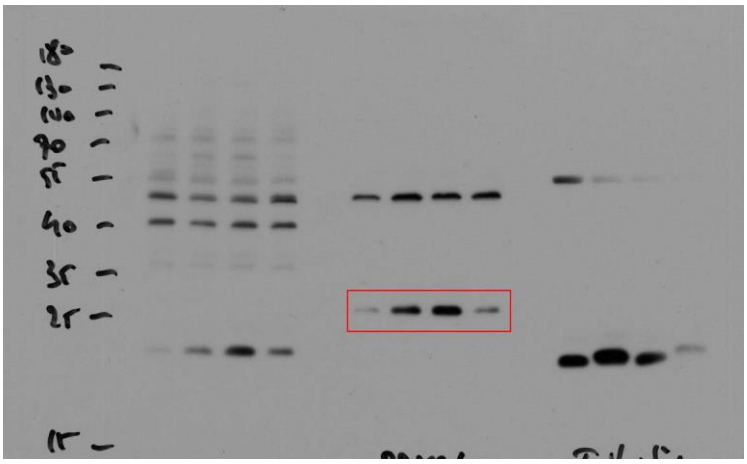

Beta5 western blot:

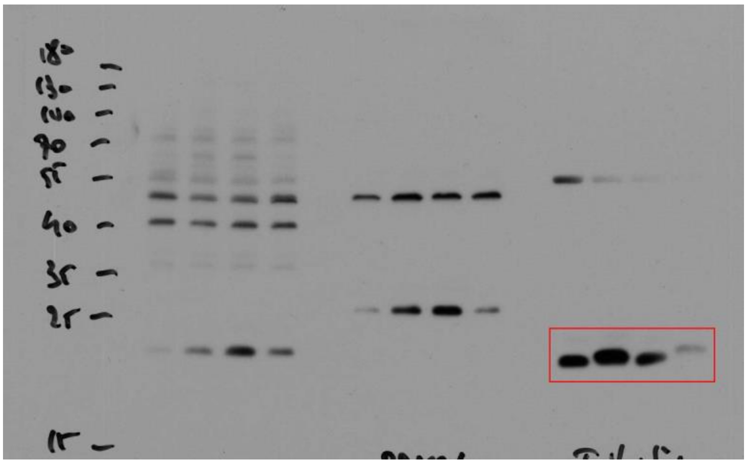

Beta5i western blot:

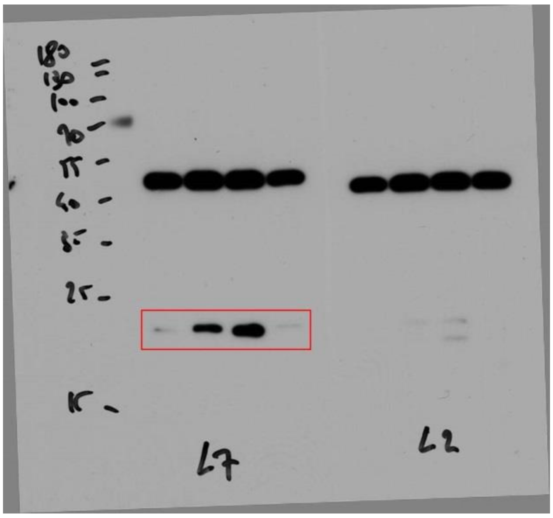

Beta1i western Blot:

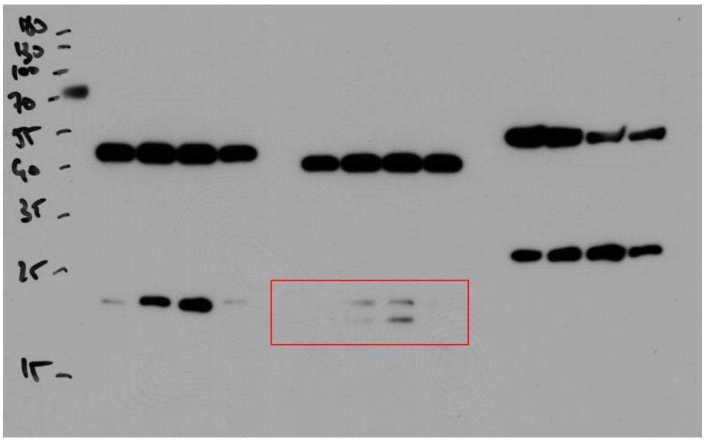

α-Tubulin western blot:

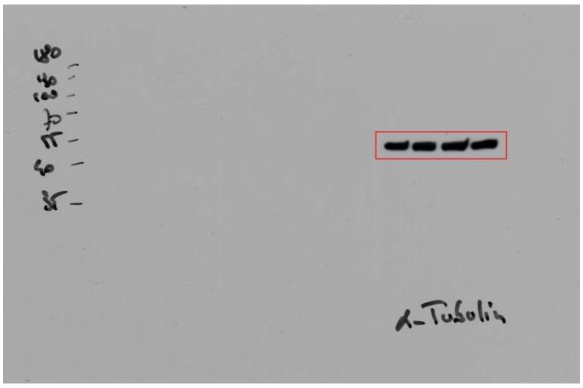

Supplement: Supplementary file 7 — Source Data for Figure 4 [file EMMM-12-e11861-s005.pdf]
